# In Vitro Evaluation of Probiotic Activities and Anti-Obesity Effects of *Enterococcus faecalis* EF-1 in Mice Fed a High-Fat Diet

**DOI:** 10.3390/foods13244095

**Published:** 2024-12-18

**Authors:** Hongying Cai, Qingya Wang, Xiling Han, Haiou Zhang, Na Wang, Yuyin Huang, Peilong Yang, Rui Zhang, Kun Meng

**Affiliations:** 1 Key Laboratory of Feed Biotechnology of Ministry of Agriculture and Rural Affairs, Institute of Feed Research, Chinese Academy of Agricultural Sciences, Beijing 100081, China; caihongying@caas.cn (H.C.); zhanghaiou@caas.cn (H.Z.); yuyinhuang99@163.com (Y.H.); yangpeilong@caas.cn (P.Y.); 2 Key Laboratory of Yunnan for Biomass Energy and Biotechnology of Environment, Yunnan Normal University, Kunming 650500, China; 18238693320@163.com (Q.W.); 17789363681@163.com (X.H.); 18213037276@163.com (N.W.); 3 Key Laboratory of Yunnan Provincial Education Department for Plateau Characteristic Food Enzymes, Yunnan Normal University, Kunming 650500, China

**Keywords:** *Enterococcus faecalis* EF-1, probiotic activities, high-fat diet, obesity, gut microbiota, SCFAs

## Abstract

This research sought to assess the anti-obesity potential of *Enterococcus faecalis* EF-1. An extensive and robust in vitro methodology confirmed EF-1’s significant potential in combating obesity, probably due to its excellent gastrointestinal tract adaptability, cholesterol-lowering property, bile salt hydrolase activity, α-glucosidase inhibition, and fatty acid absorption ability. Moreover, EF-1 exhibited antimicrobial activity against several pathogenic strains, lacked hemolytic activity, and was sensitive to all antibiotics tested. To further investigate EF-1’s anti-obesity properties in vivo, a high-fat diet (HFD) was used to induce obesity in C57BL/6J mice. Treatment with EF-1 (2 × 10^9^ CFU/day) mitigated HFD-induced body weight gain, reduced adipose tissue weight, and preserved liver function. EF-1 also ameliorated obesity-associated microbiota imbalances, such as decreasing the *Firmicutes*/*Bacteroidetes* ratio and boosting the levels of bacteria (*Faecalibacterium*, *Mucispirillum*, *Desulfovibrio*, *Bacteroides*, and *Lachnospiraceae*_NK4A136_group), which are responsible for the generation of short-chain fatty acids (SCFAs). Concurrently, the levels of total SCFAs were elevated. Thus, following comprehensive safety and efficacy assessments in vitro and in vivo, our results demonstrate that *E. faecalis* EF-1 inhibits HFD-induced obesity through the regulation of gut microbiota and enhancing SCFA production. This strain appears to be a highly promising candidate for anti-obesity therapeutics or functional foods.

## 1. Introduction

Obesity has emerged as a global epidemic and a critical public health challenge, significantly increasing the risk of various severe diseases including type 2 diabetes mellitus (T2DM), non-alcoholic fatty liver disease (NAFLD), cardiovascular diseases, hypertension, and stroke [1], frequently attributed to sedentary behaviors and excessive caloric intake [2]. The etiology of obesity is multifaceted, involving genetic, socioeconomic, and cultural factors; existing comorbidities; and chronic pharmacotherapy, but also urbanization and lifestyle choice [3]. Obesity is further linked to numerous comorbidities that can adversely affect health and well-being [4]. Its management typically encompasses calorie restriction, physical activity enhancement, and pharmacotherapy (for example, orlistat, phentermine, and liraglutide). Although effective, these drugs cause adverse effects, including diarrhea, nausea, constipation, oily fecal spotting and urgency, influenza, hypoglycemia, headache, upper-respiratory issues, etc. [5,6]. Therefore, there is a pressing requirement to craft more efficient and safer approaches for combating obesity.

The contribution of gut microbiota to the development of obesity is well described [7,8]. Changes in diet or environment (e.g., medication and stress) may trigger gut dysbiosis, thereby promoting the expansion of pathogenic microbes that are implicated in the onset of obesity [9]. The fermentation of dietary fiber by gut *Firmicutes* and *Bacteroidetes* generates short-chain fatty acids (SCFAs) such as butyrate, propionate, and acetate. These SCFAs modulate host metabolism through their action on G protein-coupled receptors (GPCRs) present on enteroendocrine cells [10]. An elevated *Firmicutes*-to-*Bacteroidetes* ratio in the gut microbiota is characteristic of obesity [10]. Imbalances in the gut microbiota can result in increased intestinal permeability, permitting the infiltration of lipopolysaccharides (LPSs), along with other bacterial endotoxins, into the body’s systems, which may trigger inflammation and contribute to obesity [11,12,13]. Conversely, a balanced gut flora may contribute to the mitigation or prevention of obesity [14,15]. Thus, preserving the equilibrium of the gut microbiota could represent a novel strategy for the prevention and management of obesity.

Lactic acid bacteria (LAB), comprising *Lactobacillus*, *Bifidobacterium*, *Streptococcus*, and *Enterococcus*, are broadly applied as probiotics and are deemed safe microorganisms by the United States Food and Drug Administration [16,17]. These LAB must endure acidic conditions, bile, and pancreatic digestive enzymes, ultimately adhering to the colonic epithelium [18,19]. Numerous previous studies have indicated that certain LAB, notably *Lactobacilli*, possess the potential to mitigate obesity and reduce blood lipid levels [20,21]. The anti-obesity effects of LAB are mediated through various mechanisms, such as adjusting gut microbiota, enhancing the production of SCFAs, altering the expression of lipid metabolism-related genes [22,23], and mitigating inflammatory responses [24]. *Enterococcus faecalis* (*E. faecalis*), a type of LAB, possesses immunomodulatory activity [25]. *E. faecalis* AG5 enhances sensitivity to glucose, insulin, and leptin; modulates long-chain fatty acids (LCFAs); and lessens the impact of HFD-induced obesity in Wistar rats [26]. Heat-killed *E. faecalis* EF-2001 has demonstrated the ability to decrease lipid accumulation and liver injury in HFD-induced obese mice by the activation of the AMPK signaling pathway [27]. Compared to *Lactobacillus* spp., *E. faecalis*-generated myristoleic acid is a new postbiotic that offers benefits in managing obesity and related health conditions [28]. *E. faecalis* strains have been recognized in various studies for their positive impact on the amelioration of liver lipid disorders, modulation of insulin signaling pathways, and enhancement of overall metabolic balance [29]. Given the comprehensive positive outcomes shown by these studies, it appears that *enterococci* could be potential candidates for preventing and treating obesity, as well as for mitigating associated complications.

The main objective of this study was to explore the probiotic potential of *E. faecalis* EF-1, encompassing tolerance to simulated gastrointestinal fluid, bile salt hydrolase activity, cholesterol degradation, fatty acid uptake, antibacterial characteristics, hemolytic activity, and antibiotic susceptibility. Furthermore, this study investigated the anti-obesity effects of *E. faecalis* EF-1 and its potential mechanisms in mice fed a HFD.

## 2. Materials and Methods

### 2.1. Chemicals

Pepsin, sodium glycylcholate, sodium taurocholate, and de Man–Rogosa–Sharpe (MRS) medium were procured from Beijing Solarbio Science & Technology Co., Ltd. (Beijing, China). Cholesterol and α-glucosidase were sourced from Sigma Aldrich Trading Ltd. (Shanghai, China). Maintenance and high-fat diets were obtained from Beijing Keao Xieli Feed Co., Ltd. (Beijing, China). Kanamycin, tetracycline, clindamycin, gentamicin, erythromycin, chloramphenicol, ampicillin, and penicillin were purchased from Changde Bikman Biotechnology Co., Ltd. (Changde, China). Assay kits for total cholesterol (TC), triacylglycerols (TG), high-density lipoprotein cholesterol (HDL-C), low-density lipoprotein cholesterol (LDL-C), very-low-density lipoprotein cholesterol (VLDL-C), glucose (GLU), aspartate aminotransferase (AST), and alanine aminotransferase (ALT) were purchased from Wuhan Shengzhiyuan Biotechnology Co., Ltd. (Wuhan, China). Other chemicals were of the highest analytical grade and were acquired from reputable commercial suppliers.

### 2.2. Strains and Growth Media

*E. faecalis* EF-1, a strain of lactic acid bacteria, was isolated from the fecal specimen of a healthy dog, identified, and deposited in the Chinese General Microorganism Collection Center (CGMCC) with the accession number 32097. The strain was cultured using the de Man–Rogosa–Sharpe (MRS) medium, which is composed of (per liter) 20 g glucose, 5 g peptone, 4 g yeast extract, 5 g beef extract, 10 g tryptone, 2 g diammonium hydrogen citrate, 5 g sodium acetate, 2 g dipotassium phosphate (K_2_HPO_4_), 0.5 g magnesium sulfate (MgSO_4_), 0.05 g manganese sulfate (MnSO_4_), and 1.0 mL Tween 80, with the pH adjusted to 6.5. For the preparation of MRS solid medium, 2% agar was incorporated into the above formulation. Prior to experimentation, *E. faecalis* EF-1 was revitalized by inoculating it into MRS solid medium and incubating at 37 °C for 48 h in a constant temperature incubator. A single, well-isolated colony was then selected and subcultured into MRS broth medium and incubated at 37 °C for 24 h. Finally, the strain was introduced into a 50 mL volume of MRS broth medium with a 2% inoculum concentration and cultured at 37 °C for a duration of 24 h to prepare the subsequent experiments procedures.

The pathogenic bacteria *Staphylococcus aureus* ATCC430, *Salmonella enterica* ATCC14028, and *Escherichia coli* CVCC195, preserved in our laboratory, were cultivated in Luria–Bertani (LB) broth. The broth was prepared with 10 g of tryptone, 10 g of NaCl, and 5 g of yeast extract per liter, and the cultures were incubated at 37 °C with agitation until they achieved a concentration of 1 × 10^8^ CFU/mL.

### 2.3. Screening of E. faecalis EF-1 for Potential Probiotic Properties

#### 2.3.1. Simulated Gastrointestinal Tolerance

The simulated gastrointestinal tolerance of *E. faecalis* EF-1 was assessed based on the methodology of Xu et al. (2023) [30] with minor adjustments. After activating, the strain underwent triple washing with 0.01 M phosphate-buffered saline (PBS, pH 7.2). The concentration of *E. faecalis* EF-1 was adjusted to 10^9^ CFU/mL using PBS. To simulate gastric digestion, which typically endures for about 3 h, 10% of *E. faecalis* EF-1 was mixed with artificial gastric fluid and incubated at 37 °C for the same period. Colony counts of *E. faecalis* EF-1 were taken at 0, 1.5, and 3 h. Following this, 1 mL of the gastric mixture was transferred to artificial intestinal fluid and incubated at 37 °C for 8 h. Colony counts were recorded at 0, 2, 4, and 8 h during this incubation. The experiment was repeated three times to confirm the reproducibility of the results.

#### 2.3.2. Cholesterol-Reducing Rate

The ability of *E. faecalis* EF-1 to reduce cholesterol was determined by the phthalaldehyde method. *E. faecalis* EF-1, at a 2% inoculum, was cultivated in MRS–cholesterol medium, which contained, per liter, 0.1 g of cholesterol and 0.2 g of cow bile salt in MRS medium, kept at a constant temperature of 37 °C for a period of 48 h. A control was established using the MRS–cholesterol medium devoid of *E. faecalis* EF-1. Post incubation, the culture was centrifuged at 9000× *g* for 10 min to separate the supernatant. The supernatant (0.5 mL) was combined with 4.5 mL of anhydrous ethanol, allowed to stand for 10 min, and then centrifuged at 3000× *g* for 15 min. Next, 0.5 mL of the supernatant was thoroughly mixed with 0.2 mL of phthalaldehyde solution and 4.3 mL of a mixed acid solution (H_2_SO_4_/acetic acid, 1:1). The absorbance value (OD) was measured at 550 nm by an enzyme-labeled instrument after standing for 30 min. Then, the cholesterol-reducing rate was calculated as follows:

Cholesterol-reducing rate = (1 − Cs/Cc) × 100%

Formula: Cs—Cholesterol content in the fermentation supernatant (μg); Cc—Cholesterol content of the control group (μg).

#### 2.3.3. Detection of BSH Activity

The bile salt hydrolase (BSH) activity of *E. faecalis* EF-1 was evaluated using the direct plate assay method. *E. faecalis* EF-1 (20 μL) was inoculated onto round filter paper disks that had been placed on MRS agar plates, which were beforehand supplemented with 0.37% (*w*/*v*) calcium chloride and either 0.5% (*w*/*v*) sodium taurocholate hydrate (TCA) or 0.5% (*w*/*v*) sodium glycocholate hydrate (GCA). The inoculated agar plates were placed in an anaerobic environment at 37 °C for 72 h. The presence of BSH activity was evidenced by the appearance of a precipitate encircling the filter paper disks.

#### 2.3.4. Detection of Antibacterial Activity

The antibacterial properties of the *E. faecalis* EF-1 culture were assessed by utilizing the Oxford cup method. A volume of 50 μL from each pathogenic bacterial culture—*Staphylococcus aureus* ATCC430, *Salmonella enterica* ATCC14028, and *Escherichia coli* CVCC195—with an OD600 nm of 0.7 was inoculated onto freshly prepared LB agar plates containing 2% agar (*w*/*v*). Subsequently, 100 μL of the *E. faecalis* EF-1 culture was placed into a 6 mm Oxford cup that had been pre-drilled into the LB agar plates. The plates were incubated at 37 °C for 24 h, after which the presence and size of any inhibition zones were observed and measured.

#### 2.3.5. The Inhibition Activity of α-Glucosidase

*E. faecalis* EF-1 was cultured in a 50 mL volume of MRS broth medium at a 2% inoculum ratio and incubated at 37 °C for 24 h. The culture was then centrifuged at 8000× *g* for 5 min, after which the supernatant was carefully collected. The assessment of α-glucosidase activity was assessed following the guidelines provided by Apostolidis et al. (2023), with minor adjustments [31]. A reaction mixture was formulated by combining 50 μL of supernatant with 100 μL of 0.1 M phosphate buffer solution at pH 6.9 that included α-glucosidase at a concentration of 1.0 U/mL, and this mixture was then incubated in a 96-well plate at 25 °C for a duration of 10 min. After the preincubation period, 50 μL of 5 mM *p*-nitrophenyl-α-*D*-glucopyranoside solution, which was prepared in 0.1 M phosphate buffer at pH 6.9, was introduced into each well. An additional 5 min incubation at 25 °C was applied to the reaction mixtures. Optical density measurements at 405 nm were taken both prior to and following the incubation, with comparisons made to a control that included 50 μL of buffer solution instead of the supernatant. The percentage inhibition of α-glucosidase activity was calculated using the following formula:

%inhibition = (1 − Cs/Cc) × 100

Formula: Cs—α-glucosidase activity in fermentation supernatant; Cc—α-glucosidase activity of the control group.

#### 2.3.6. Fatty Acid Absorption Assay

The assay for fatty acid absorption was conducted according to a previous study [32]. In brief, *E. faecalis* EF-1 was inoculated into 50 mL of MRS broth medium, supplemented with 0.5% (*w*/*v*) Brij58 and 0.25 mmol/L sodium palmitate at a 2% inoculum ratio, and incubated at 37 °C for 24 h. The concentration of fatty acids in the culture medium was measured using a free fatty acid assay kit.

#### 2.3.7. Measurement of Antibiotic Resistance Phenotypes

Antibiotic resistance phenotypes of *E. faecalis* EF-1 were identified using the disk diffusion susceptibility test. After inoculating 100 μL of *E. faecalis* EF-1 onto the agar plate, antibiotic disks containing the following antibiotics were applied: kanamycin (30 μg/disk), tetracycline (30 μg/disk), clindamycin (2 μg/disk), gentamicin (10 μg/disk), erythromycin (15 μg/disk), chloramphenicol (30 μg/disk), ampicillin (10 μg/disk), and penicillin (10 μg/disk). Subsequently, the plates were placed in an incubator at 37 °C for a period of 24 h to assess the susceptibility of *E. faecalis* EF-1. The presence and size of the inhibitory zones surrounding the antibiotic disks were used to determine the resistance breakpoints for various antibiotics, following established guidelines [33].

#### 2.3.8. Hemolytic Activity

To assess the hemolytic activity of *E. faecalis* EF-1, the activated strain was cultured on blood agar plates supplemented with 5% (*w*/*v*) sheep blood and incubated at 37 °C for a period of 48 h. Following this incubation period, the presence of hemolysis zones was examined. *E. coli* CVCC195 served as a positive control for comparison.

### 2.4. Animals Experiment Design

A total of thirty-two male mice, aged 7 weeks and weighing 22 ± 2 g, with a C57BL/6J genetic background, were sourced from Vital River Laboratory Animal Technology Co., Ltd. (Beijing, China). The animals were housed in standard laboratory conditions, which included a room temperature of 22 ± 2 °C, a relative humidity of 55 ± 5%, and a 12:12 h light–dark cycle. Before the commencement of the experiment, the mice were allowed unrestricted access to a regular chow diet and water for a week to acclimate. Subsequently, the mice were distributed randomly across four distinct groups: the control group (CT group, *n* = 8), which was fed a maintenance diet; the model group (HFD group, *n* = 8), which was given a HFD (#D12492; 60% kcal from fat; Research Diets); the positive control group (PC group, *n* = 8), which was administered atorvastatin calcium at a dose of 30 mg/kg in 0.2 mL of sterilized saline (0.9%) via gavage; and the *E. faecalis* EF-1 group (EF-1 group, n = 8), which received the HFD diet with the addition of 0.2 mL of *E. faecalis* EF-1 (1 × 10^10^ CFU/mL). Two mice were housed per cage. The moderate dose of 2 × 10^9^ CFU/day was selected based on previous studies that evaluated the effects of probiotics on mice through intragastric administration [34,35]. *E. faecalis* AG5 mitigated HFD-induced obesity in Wistar rats at a dose of 1.1 × 10^9^ CFU/day [26]. *E. faecalis* ATCC19433, at a dose of 10^9^ CFU/day, exerted a hypocholesterolemic effect on hypercholesterolemic mice [36]. The dose of atorvastatin calcium was determined according to the previous report [37]. Sterilized saline in an equivalent volume was gavaged to the CT and HFD groups. The intervention time of the mice was 16 weeks, and food intake, remaining food, and weight were recorded once a week. The animal experiments were performed in accordance to the program and license (authorization number: IFR-CAAS20240415) approved by the Committee of the Feed Research Institute of Chinese Academy of Agricultural Sciences (CAAS).

### 2.5. Sample Collection

At the termination of the experiment, a 16 h fast was imposed on all mice, after which they were sacrificed to collect blood, livers, white adipose tissues (WATs), spleens, kidneys, and cecal contents. Serum was extracted by centrifuging the blood samples at 3000× *g* at 4 °C for a duration of 10 min, and then it was kept at −80 °C for subsequent analysis. Cecal contents were collected on ice and immediately stored in liquid nitrogen until 16S rRNA sequencing. The liver, WATs, spleen, and kidneys were weighed immediately and recorded. Some liver and WATs samples were immersed in 4% neutral buffered paraformaldehyde for morphological analysis, and the leftover samples were promptly frozen in liquid nitrogen and conserved at −80 °C.

### 2.6. Biochemical Assay of Serum and Liver Tissues

Following the cleansing of liver tissue with ice-cold phosphate-buffered saline (PBS, pH 7.4), a 0.1 g sample of liver tissue was weighed out in 0.9 mL of PBS and homogenized using a homogenizer, and then centrifugation at 3000× *g* at 4 °C for a duration of 15 min was performed to separate the supernatant for the determination of biochemical parameters. The serum and liver concentrations of TC, TG, HDL-C, LDL-C, VLDL-C, ALT, AST, and GLU were quantified. All procedures were conducted in strict accordance with the manufacturers’ instructions provided with the kits.

### 2.7. Histological Evaluation

#### 2.7.1. Oil Red O Staining of Liver Tissue

The liver samples, preserved in 4% neutral buffered paraformaldehyde, were processed for histological examination. They were first dehydrated and then embedded in a suitable medium, followed by sectioning at a thickness of 4–5 μm. The sections were subsequently stained with Oil Red O solution to visualize lipid content. After staining, the sections were differentiated in 60% isopropyl alcohol and then counterstained with hematoxylin. Finally, the sections were mounted with glycerin gelatin, and observations were made using a microscope (NIKON ECLIPSE E100, NIKON, Tokyo, Japan) and an imaging system (NIKON DS-U3, NIKON, Tokyo, Japan).

#### 2.7.2. Hematoxylin and Eosin Staining of Liver and Adipose Tissue

Liver and adipose tissue samples, once fixed in formalin, underwent dehydration and were embedded in paraffin wax. Tissue sections, 4–5 μm thick, were prepared from the liver tissue and stained with hematoxylin and eosin (H&E), which were then scrutinized under a 400 × microscope to perform an in-depth examination.

### 2.8. 16S rRNA Sequencing and Processing of Gut Microbiota

To discern differences in microbial composition across various experimental groups, a total of 20 samples of cecal content were gathered for 16S rRNA gene amplicon sequencing utilizing the Illumina NovaSeq platform at Beijing Biomarker Technologies Co., Ltd. (Beijing, China). DNA extraction from the cecal contents was performed using the TGuide S96 Magnetic Stool DNA Kit from Tiangen Biotech (Beijing) Co., Ltd. (Beijing, China), following the manufacturer’s protocol. The V3-V4 hypervariable region of the 16S rRNA gene was targeted for amplification using the universal primers 338F and 806R. After PCR amplification, the amplicons were cleaned with Agencourt AMPure XP Beads (Beckman Coulter, Indianapolis, IN, USA), and their quantities were measured using the Qubit dsDNA HS Assay Kit and Qubit 4.0 Fluorometer (Invitrogen, Thermo Fisher Scientific, Oregon, Waltham, USA). After determining the quantity, the amplicons were pooled to have the same molar concentration. The subsequent library construction and sequencing were performed on the Illumina NovaSeq 6000 platform (Illumina, San Diego, CA, USA). The complete set of raw data have been made available in the NCBI Sequence Read Archive, identified by the accession number SRP543468.

The dada2 method, as referenced in [38], was implemented within QIIME2 version 2020.6 [39] for the processes of denoising, merging paired-end sequences, and eliminating chimeric sequences to derive amplicon sequence variants (ASVs). The alpha diversity indices were computed using QIIME2 and visualized with R software (version 3.1.1). Bacterial diversity was quantified using the Chao1, ACE, Shannon, and Simpson indices. Beta diversity calculations were performed to evaluate the similarities and differences among microbial communities in various samples. Principal coordinate analysis (PCoA) was employed to interpret the beta diversity results.

### 2.9. Determination of SCFA Production in HFD-Induced Obese Mice

We transferred a sample of cecum contents into a 2 mL Eppendorf (EP) tube and extracted it with addition of 1 mL of distilled water, and then we vortexed it for 10 s to ensure proper extraction. We placed the sample in a ball mill and processed it by homogenization at a frequency of 40 Hz for 4 min, then subjected it to ultrasonic treatment for 5 min while incubating in an ice bath, repeating this process three times. We centrifuged the sample at 5000× *g* at 4 °C for 20 min. We aspirated 0.8 mL of the supernatant and transferred it into a fresh 2 mL EP tube. We added 0.1 mL of 50% H_2_SO_4_ and 0.8 mL of the extraction solution (containing 25 mg/L as an internal standard in methyl tert-butyl ether), vortex mixed for 10 s, and then oscillated for 10 min, followed by another 10 min of ultrasonic treatment while incubating in an ice bath. We subjected the sample to centrifugation once more at 10,000× *g* and 4 °C for 15 min, then placed it in storage at −20 °C for 30 min. Finally, we carefully moved the supernatant to a clean 2 mL glass vial for subsequent gas chromatography–mass spectrometry (GC-MS) analysis.

The Shimadzu GC2030-QP2020 NX gas chromatography–mass spectrometer system was employed, equipped with a high-polarity fused silica capillary column (HPFFAP). For the injection, 1 μL of the analyte was used in split mode, maintaining a split ratio of 5:1. As the carrier gas, helium was employed, featuring a front inlet purge flow rate of 3 mL/min and a column flow rate of 1.2 mL/min. The initial temperature setting was 50 °C, kept for 1 min, followed by a ramp up to 150 °C at a speed of 50 °C per minute, and then held at that temperature for 1 min. Subsequently, the temperature profile included raising the temperature to 170 °C at a rate of 10 °C per minute for 0 min, then to 225 °C at a rate of 25 °C per minute for 1 min, and ultimately to 240 °C at a rate of 40 °C per minute for 1 min. Meanwhile, the temperatures for the injection port, transfer line, quadrupole, and ion source were adjusted to 220 °C, 240 °C, 150 °C, and 240 °C, respectively. In electron impact mode, the ionization energy was configured at −70 eV. The acquisition of mass spectrometry data was executed in Scan/SIM mode, with a mass-to-charge (*m*/*z*) range of 33–150, following a 3.75 min solvent delay.

### 2.10. Statistical Analysis

The presented data indicate the mean values ± standard deviation (SD). Data analysis and organization were carried out with GraphPad Prism version 10.2.3. Student’s *t*-test was the method chosen to compare statistical disparities between groups, with a *p*-value threshold of less than 0.05 considered to indicate statistical significance.

## 3. Results

### 3.1. Potential Probiotic Properties

#### 3.1.1. Tolerance of *E. faecalis* EF-1 to Gastric and Intestinal Juices

*E. faecalis* EF-1 was tested for viability in both artificial gastric juice and intestinal juice, with the results showing growth in these environments (Table 1). Specifically, the survival rates of *E. faecalis* EF-1 were calculated as 109.68% in the simulated gastric juice and 890.35% in intestinal fluid.

#### 3.1.2. Cholesterol-Reducing Capacity of *E. faecalis* EF-1

After culturing *E. faecalis* EF-1 in MRS medium supplemented with cholesterol, the results demonstrated that *E. faecalis* EF-1 exhibited a significant cholesterol-lowering capability, achieving a cholesterol-reducing rate of 48.69%.

#### 3.1.3. BSH Activity

**Figure 1:**
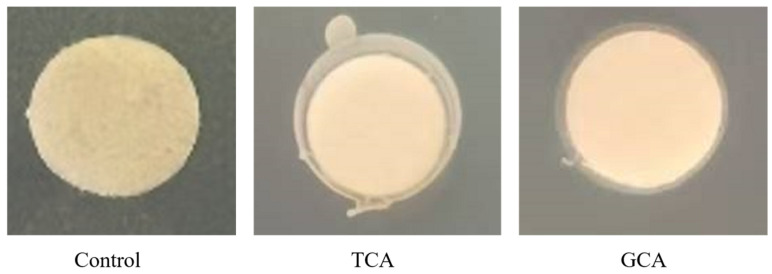
Visual representation of BSH acidity of *E. faecalis* EF-1.

The effects of *E. faecalis* EF-1 on sodium taurocholate hydrate (TCA) and sodium glycocholate hydrate (GCA) were evaluated. Figure 1 illustrates a ring of precipitate encircling the filter paper on MRS agar plates amended with either TCA or GCA. The findings suggest that *E. faecalis* EF-1 possessed both TCA and GCA hydrolytic activity.

#### 3.1.4. Fatty Acid Absorption by *E. faecalis* EF-1

*E. faecalis* EF-1’s fatty acid uptake was tested by incubating the strain in MRS broth enriched with 0.25 mmol/L sodium palmitate for a duration of 24 h. Post incubation, the broth was analyzed to measure the remaining fatty acid levels. The findings revealed that the addition of *E. faecalis* EF-1 significantly reduced the total fatty acid content in the medium, demonstrating an absorption capacity of 59%.

#### 3.1.5. The Inhibition Effect of α-Glucosidase

The fermentation supernatant from *E. faecalis* EF-1 was utilized to assess its inhibitory effect on α-glucosidase activity. The findings indicated that *E. faecalis* EF-1 exhibits an inhibitory rate of 38.51% against α-glucosidase activity.

#### 3.1.6. Measurement of Antibacterial Activity of *E. faecalis* EF-1

*E. faecalis* EF-1 was evaluated for its ability to form inhibitory zones against *S. aureus* ATCC430, *S. enterica* ATCC14028, and *E. coli* CVCC195. The antibacterial zone diameters of *E. faecalis* EF-1 against Gram-positive bacteria *S. aureus* ATCC430 reached 13.01 mm, indicating a marked discrepancy in comparison to the control group. The antibacterial zone diameters for the Gram-negative bacteria *S. enterica* ATCC14028 and *E. coli* CVCC195 were 13.76 mm and 11.76 mm, respectively.

#### 3.1.7. Antibiotic Resistance

The susceptibility of *E. faecalis* EF-1 to antibiotics was assessed, revealing that it was sensitive to all the antibiotics under evaluation, encompassing kanamycin, tetracycline, clindamycin, gentamicin, erythromycin, chloramphenicol, ampicillin, and penicillin.

#### 3.1.8. Hemolytic Activity of *E. faecalis* EF-1

**Figure 2:**
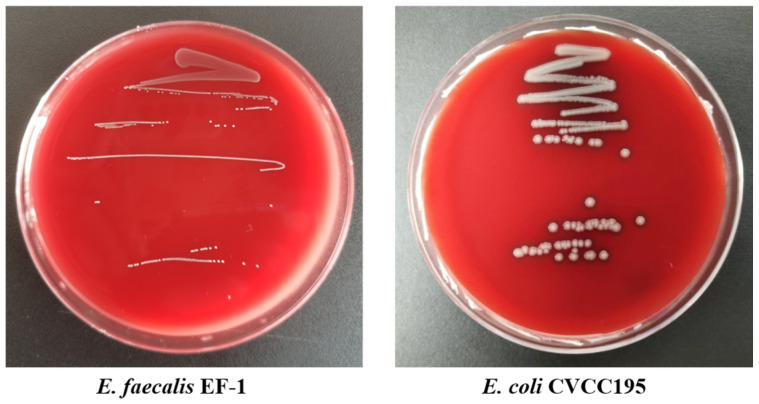
Detection result of hemolysis test.

*E. faecalis* EF-1 demonstrated γ-hemolytic activity, characterized by the absence of hemolysis, when grown on blood agar, as depicted in Figure 2.

### 3.2. E. faecalis EF-1 Alleviated HFD-Induced Obesity in Mice

#### 3.2.1. *E. faecalis* EF-1 Reduced Body Weight in HFD-Induced Obese Mice

**Figure 3:**
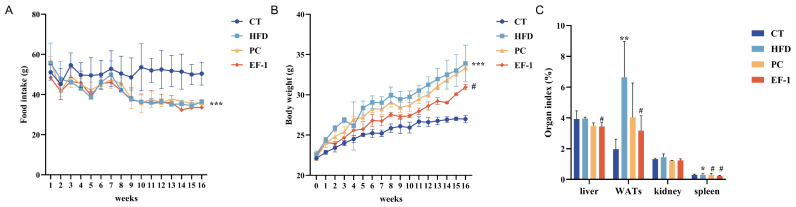
The effect of *E. faecalis* EF-1 intervention on HFD-induced obese mice. (**A**) The average food intake across the weeks. (**B**) Body weight variations throughout the weeks. (**C**) The organ index (liver, white adipose tissue, kidney, and spleen). CT refers to the control group, HFD to the high-fat diet group, PC to the positive group, and EF-1 to the *E. faecalis* EF-1 intervention group. *n* = 8 mice per group. The symbols *, **, and *** indicate significant differences at the *p* < 0.05, *p* < 0.01, and *p* < 0.001 levels, respectively, when comparing the HFD and CT groups. Similarly, the symbol # denotes significant differences at *p* < 0.05 for the comparison between the EF-1 and HFD groups or between the PC and HFD groups.

In vitro experiments substantiated the idea that *E. faecalis* EF-1 possesses the potential to regulate lipid metabolism. To validate these results, in vivo validation was performed using a mouse model. As illustrated in Figure 3A, compared to the CT group, food intake in the HFD group was significantly reduced (*p* < 0.05). There was no significant difference between EF-1 and HFD groups. After 16 weeks of HFD consumption, there was a significant increase in body weight in the HFD group compared to the CT group. As shown in Figure 3B, the body weight in the *E. faecalis* EF-1 intervention group was significantly decreased (*p* < 0.05), which was better than the inhibitory effect of the PC group. These significant differences persisted through week 16, underscoring the potent influence of *E. faecalis* EF-1 in preventing body weight. This suggests that the reduction in body weight attributed to *E. faecalis* EF-1 was not due to decreased food consumption.

Changes in body weight were also mirrored in the weight alterations of various organs and tissues. Consequently, the impact of *E. faecalis* EF-1 on fat deposition was assessed by monitoring changes in the weights of the liver, white adipose tissues (WATs), kidneys, and spleen in obese mice. Figure 3C illustrates that, in comparison to the CT group, indices of WATs and spleen were significantly higher (*p* < 0.05) in the HFD group. Notably, the WAT and spleen weights were significantly lower in the EF-1 group (*p* < 0.05). Moreover, liver index was significantly reduced after *E. faecalis* EF-1 intervention compared to the HFD group. The index of the kidneys did not show significant differences among four groups. The intervention with *E. faecalis* EF-1 prevented the enlargement of WATs and spleen, akin to the PC group, suggesting that the significant reduction in body weight involved a decrease in various tissues.

#### 3.2.2. *E. faecalis* EF-1 Prevented Lipid Accumulation and Reduced Liver and WAT Damage in Obese Mice

**Figure 4:**
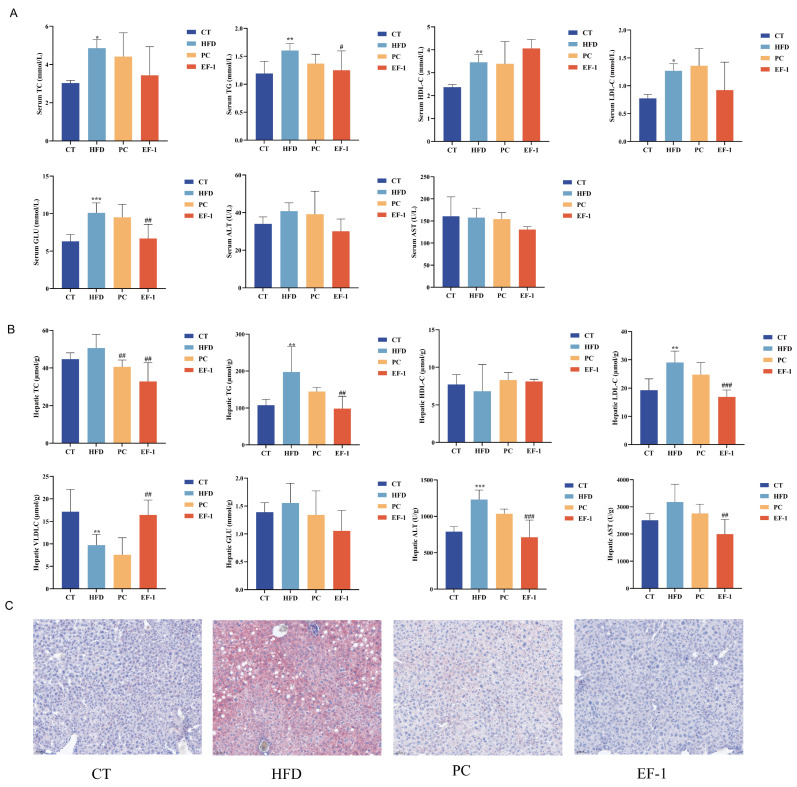
*E. faecalis* EF-1 alleviated serum and liver biochemical indices in obese mice fed a HFD. (**A**) The role of *E. faecalis* EF-1 in altering serum biochemical indices. (**B**) The role of *E. faecalis* EF-1 in altering liver biochemical indices. (**C**) The observation of liver tissues stained with Oil Red O, with a scale reference of 100 μm. *n* = 8 mice per group. The symbols *, **, and *** indicate significant differences at the *p* < 0.05, *p* < 0.01, and *p* < 0.001 levels, respectively, when comparing the HFD and CT groups. Similarly, the symbols #, ##, and ### denote significant differences at the same significance levels for the comparison between the EF-1 and HFD groups or between the PC and HFD groups.

As shown in Figure 4A, compared to the CT group, the serum levels of TC, TG, HDL-C, LDL-C, and GLU were significantly elevated (*p* < 0.05) in the HFD group. The *E. faecalis* EF-1 intervention significantly reduced the levels of TG and GLU (*p* < 0.05) and lowered the levels of TC, LDL-C, ALT, and AST with no significant difference, having a better regulatory effect than the PC group. As shown in Figure 4B, compared to the CT group, hepatic levels of TG, ALT and GLU were significantly elevated (*p* < 0.05), and VLDL-C was significantly decreased (*p* < 0.05). *E. faecalis* EF-1 intervention significantly reduced TC, TG, LDL-C, ALT, and AST (*p* < 0.05) and heightened the level of VLDL-C (*p* < 0.05), which had a better regulatory effect than the PC group. Furthermore, the staining of liver tissues with Oil Red O revealed that the EF group exhibited less lipid accumulation compared to the HFD group (Figure 4C).

#### 3.2.3. *E. faecalis* EF-1 Prevents the Liver and WAT Damage

**Figure 5:**
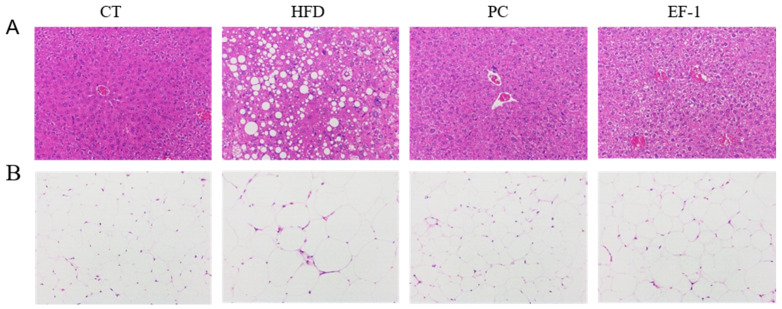
*E. faecalis* EF-1 intervention alleviated the pathological state of the liver and WATs in mice fed a HFD. H&E staining of liver tissue (**A**) and WATs (**B**), with a scale reference of 100 μm. *n* = 4 mice per group.

Figure 5A demonstrates that in comparison to the CT group, liver sections from obese mice exhibited disrupted lobular architecture, blurred cellular boundaries, extensive damage, and numerous vacuoles of varying sizes. Notably, *E. faecalis* EF-1 treatment resulted in a significant reduction in hepatic steatosis. Similarly, adipocytes in the HFD group were larger compared to those in the CT group; however, these changes were mitigated following *E. faecalis* EF-1 treatment (Figure 5B).

#### 3.2.4. *E. faecalis* EF-1 Regulated the Structure and Composition of the Gut Microbiota in Obese Mice

**Figure 6:**
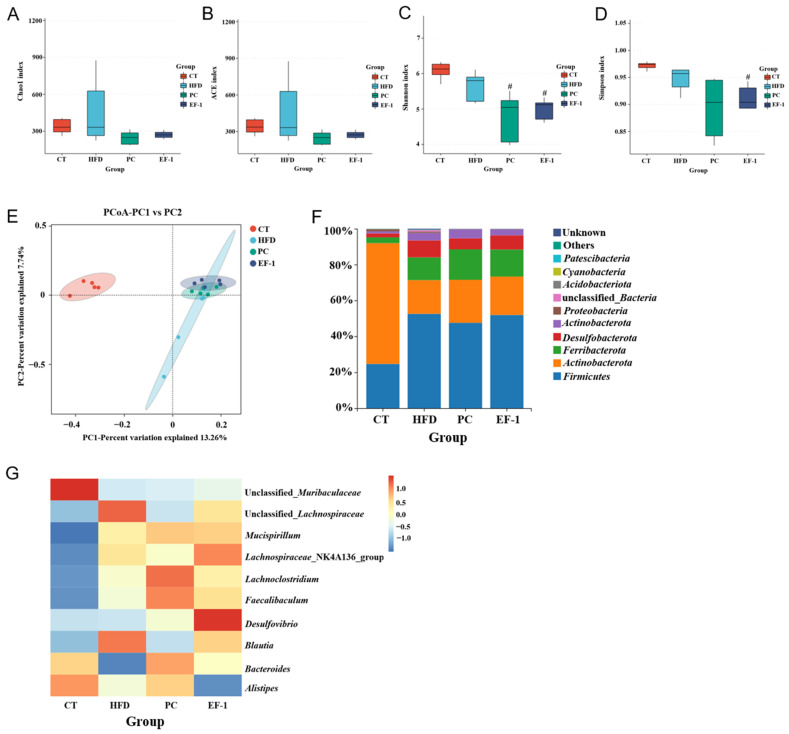
The effect of *E. faecalis* EF-1 on the structure of gut microbiota. (**A**) The Chao 1 index. (**B**) The ACE index. (**C**) The Simpson index. (**D**) The Shannon index. (**E**) PCoA analysis. (**F**) The relative abundance of gut microbiota at the phylum level. (**G**) A heatmap displaying the hierarchical clustering of bacterial genera profiles at the genus level. *n* = 5 mice per group. Differences that are significant at the *p* < 0.05 level between EF-1 and HFD groups are marked with #.

To investigate the impact of *E. faecalis* EF-1 on gut microbiota in mice consuming a HFD, we conducted 16S rRNA gene sequencing to assess the compositions of the microbial community across different groups. As depicted in Figure 6, no significant differences were detected among the CT, HFD, EF-1, and PC groups for the Chao1 and ACE indices (Figure 6A,B), indicating equivalent species richness in their gut microbiota. Analogously, for the Simpson and Shannon indices (Figure 6C,D), no significant differences were found among the four groups, implying similar species diversity in their gut microbiota. To further examine the compositional variations within the gut microbiota among the groups, PCoA was applied. Figure 6E reveals a pronounced separation between the HFD and CT groups, highlighting the significant alterations in gut microbiota composition due to a high-fat diet. The EF-1 and PC groups exhibited similar clustering patterns in their microbiota compositions but were distinctly differentiated from the HFD group, suggesting that the administration of *E. faecalis* EF-1 had a significant influence on the microbial composition clustering.

An in-depth analysis of bacterial species distribution was performed across the four groups at various taxonomic levels. In the gut microbiota of mice at the phylum level, as depicted in Figure 6F, *Firmicutes*, *Bacteroidota*, *Deferribacterota*, *Desulfobacterota*, and *Actinobacteriota* were identified as the predominant bacterial groups across the four groups. The abundance of *Firmicutes* was markedly higher, while *Bacteroidota* was considerably lower in the HFD group compared to the CT group, indicating that a HFD can disrupt the gut microbiota balance in mice. Conversely, after *E. faecalis* EF-1 intervention, the abundance of *Firmicutes* was reduced, and *Bacteroidota* was elevated compared to the HFD group, but there was no significant difference between the EF-1 and HFD groups. The EF-1 group showed a decreased *Firmicutes*/*Bacteroidota* ratio, which was more closely aligned with the CT group, suggesting that *E. faecalis* EF-1 mitigated the gut microbiota imbalance associated with a high-fat diet.

Subsequently, we conducted an analysis of the genus-level composition of the microbiota across the four groups, as presented in Figure 6G. In comparison to the CT group, the relative abundances of *Faecalibaculum*, *Mucispirillum*, *Lachnospiraceae*_NK4A13_group, and *Blautia* were found to be increased, whereas the relative abundances of unclassified_*Muribacuaceae*, *Bacteroides*, and *Alistipes* were decreased. The administration of *E. faecalis* EF-1 resulted in increases in the relative abundances of *Faecalibaculum*, *Mucispirillum*, *Desulfovibrio*, *Bacteroides*, and *Lachnospiraceae*_NK4A13_group, which were akin to the composition of the gut microbiota observed in the PC group.

#### 3.2.5. *E. faecalis* EF-1 Enhanced SCFA Production in HFD-Induced Obese Mice

**Figure 7:**
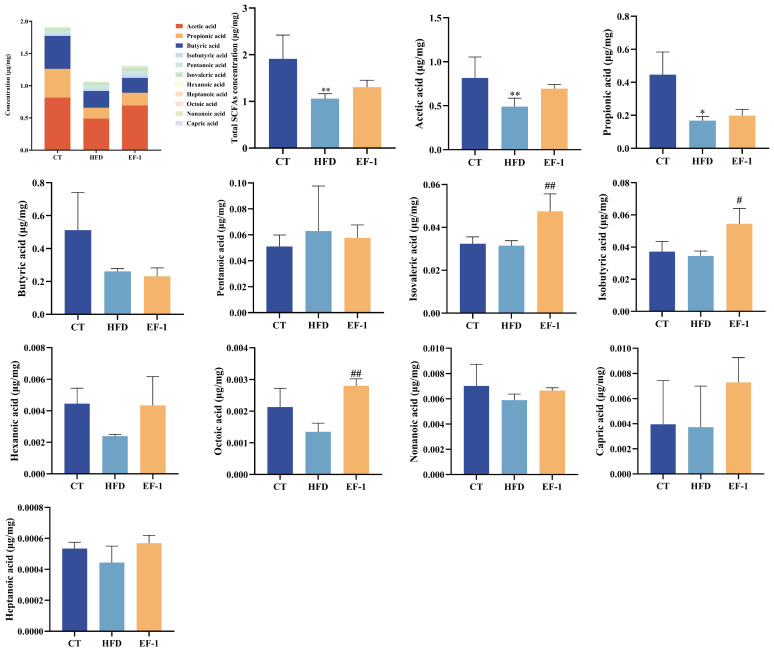
Effects of *E. faecalis* EF-1 intervention on SCFA production. *n* = 5 mice per group. The symbols * and ** indicate significant differences at *p* < 0.05 and *p* < 0.01, respectively, when comparing HFD and CT groups. Similarly, the symbols # and ## denote significant differences at the same significance levels for the comparison between the EF-1 and HFD groups.

As depicted in Figure 7, the total SCFA concentration in the CT group was 1.91 μg/mg, which was reduced to 1.06 μg/mg in the HFD group and increased to 1.30 μg/mg following intervention with *E. faecalis* EF-1, although this increase was not statistically significant. Specifically, compared to the CT group, the levels of SCFAs such as acetic acid, propionic acid, butyric acid, nonanoic acid, hexanoic acid, heptanoic acid, isobutyric acid, isovaleric acid, capric acid, and octoic acid decreased, while the level of pentanoic acid was elevated. Nevertheless, EF-1 intervention led to increases in the levels of all tested SCFAs, except for butyric acid.

## 4. Discussion

The current study investigated the potential probiotic characteristics and confirmed the lipid-lowering effect of *E. faecalis* EF-1 in vitro. *E. faecalis* EF-1 demonstrated a significant cholesterol-reducing rate (48.69%) that probably occurs through co-precipitation [40], assimilation, and absorption [41], but that process is complex and not yet fully elucidated. One potential mechanism involves the ability of probiotics to deconjugate bile salts. *E. faecalis* EF-1 bacterial suspension exhibited BSH activity, including activity against sodium taurocholate and sodium glycocholate (Figure 1). BSH activity facilitates the conversion of bile salts into amino acids and free bile acids, capable of forming complexes with cholesterol, thereby lowering cholesterol levels [42,43]. *E. faecalis* EF-1 showed potent α-glucosidase inhibitory activity (38.51%), comparable to *Lactobacillus* strains with reported values ranging from 35% to 60% by Oh et al. (2018) [44], and less effective than the α-glucosidase inhibitory activity (>75%) of four specific strains: *Lactiplantibacillus plantarum* MG4229, MG4296, MG5025, and *Lacticaseibacillus paracasei* MG5012 [45]. The inhibition of α-glucosidase, an enzyme that catalyzes the final step in the digestion of polysaccharides, slows the release and absorption of post-meal glucose, thus preventing postprandial hyperglycemia, delaying the metabolism of carbohydrate, and preventing the overconsumption of glucose [45]. *E. faecalis* EF-1, with its potential α-glucosidase inhibitory activity, could be a novel probiotic candidate for lowering blood glucose. Excess sugar is converted into fatty acids, enhancing triglyceride production and leading to adipocyte accumulation [46]. In this study, the fatty acid absorption capacity of *E. faecalis* EF-1 was 59%. Studies have indicated that LAB have the capacity to decrease fat levels, and most probiotics can suppress the proliferation of pathogenic intestinal bacteria [47]. *E. faecalis* EF-1 demonstrated inhibitory effects on certain Gram-negative and Gram-positive pathogenic bacteria. Moreover, for probiotics to be effective in the intestines, they must first tolerate the harsh environment of the upper gastrointestinal tract. *E. faecalis* EF-1 showed significant tolerance to simulated gastrointestinal conditions, with survival rates of 109.68% and 890.35%, respectively. Furthermore, in accordance with the guidelines for using LAB, each strain must undergo a safety evaluation before use [48]. Experiments on antibiotic resistance and hemolytic activity confirmed that *E. faecalis* EF-1 was sensitive to clinically used antibiotics, showing no resistance, and lacked hemolytic activity. Therefore, *E. faecalis* EF-1 exhibited favorable anti-obesity properties, good gastrointestinal adaptability, and antimicrobial activity, without antibiotic resistance or hemolytic activity in vitro.

The impact of *E. faecalis* EF-1 on obesity induced by a HFD was evaluated. *E. faecalis* EF-1, administered at a dosage of 2 × 10^9^ CFU/day, was orally provided to mice alongside the HFD for a duration of 16 weeks. This intervention with *E. faecalis* EF-1 was observed to mitigate excessive weight gain associated with a HFD. Our results corroborate the findings from earlier investigations, which demonstrated that *E. faecalis* AG5 and *E. faecalis* FK-23 also ameliorate obesity induced by high-fat diets [26,49]. Organ indices serve as biomarkers of an animal’s physiological health, with chronic high-fat diets being potentially detrimental, leading to liver and kidney malfunction, inflammation, and hypertrophy [50]. The reduction in body weight gain is likely attributed to the decreased weights of WATs, liver, and spleen. Our findings indicate that *E. faecalis* EF-1 modestly reduced body weight gain, affecting multiple organs, and could effectively mitigate the damage to the liver and WATs. Concurrently, mice in the HFD group displayed large intracytoplasmic vacuoles in their liver, suggesting a moderate fatty liver condition. HFD also induced adipocyte hypertrophy, a dysfunction that fails to manage excess fat [51]. The intervention with *E. faecalis* EF-1 alleviated the pathological states of the liver and WATs in obese mice. *E. faecalis* EF-1 intervention led to a reduction in lipid accumulation, including TC, TG, and LDL-C in the liver and serum, compared to the HFD group. Our findings align with a study that *E. faecalis* WEFA23 lowered the serum levels of TC, TG, and LDL [52]. The combination of *E. faecium* R0026 and *Bacillus subtilis* R0179 markedly reduced serum levels of TG, TC, LDL-C and HDL-C, as well as hepatic TC, exhibiting a cholesterol-lowering effect of 46% under low-cholesterol concentration conditions and 58% under high-cholesterol concentration conditions in an in vitro experiment [53]. A plausible explanation for this reduction is the cholesterol-lowering ability, BSH activity, and fatty acid intake ability of *E. faecalis* EF-1. The observed decrease in GLU levels in serum and liver may be attributed to the α-glucosidase inhibitory activity. The presence of AST and ALT in serum can serve as indicators of liver function or injury, with the liver being the primary organ responsible for lipid metabolism [54]. The intervention with *E. faecalis* EF-1 lowered the levels of ALT and AST in the liver and serum, although the differences did not reach statistical significance.

*E. faecalis* EF-1 intervention influenced the gut microbiota composition in mice consuming a HFD. As depicted in Figure 6F, the mice on a HFD had a higher *Firmicutes*-to-*Bacteroidetes* (F/B) ratio in their gut microbiota compared to the CT group, which is positively correlated with body mass index (BMI) and tends to be elevated in individuals with obesity [55]. However, treatment with *E. faecalis* EF-1 reduced the F/B ratio, suggesting that it could alleviate gut microbiota dysbiosis induced by a HFD. *E. faecalis* EF-1 up-regulated the SCFA-producing bacteria, including *Bacteroides*, *Faecalibaculum*, *Mucispirillum, Desulfovibrio*, and *Lachnospiraceae*_NK4A136_group compared to the HFD group. The result is consistent with the fact that *E. faecalis* SF68 enriched the microbes (i.e., *Bifidobacterium*, *Akkermansia*, and *Faecalibacterium*) associated with SCFA production in obese mice [56]. Studies have indicated that *Bacteroides* can prevent obesity and insulin resistance [57]. *Faecalibaculum* is well recognized for its capacity to break down non-digestible dietary fibers, including carbohydrates such as starch, that bypass absorption in the host’s small intestine [58,59]. *Mucispirillum* is believed to be involved in energy metabolism and to play a role in the production of free fatty acids in conjunction with SCFAs [60]. *Desulfovibrio*, recognized as a potential acetic acid producer, has been found to have substantial anti-NAFLD impacts in HFD-fed mice [61]. *Lachnospiraceae*_NK4A136_group, classified as butyrate-producing bacteria, has demonstrated the preservation of gut barrier integrity and exhibit a negative correlation with intestinal permeability in mice [62], as well as enhanced gut barrier function [63].

Previous research has demonstrated that SCFAs serve as an energy source for the cells lining the intestine and also function as energy and signaling molecules within the circulatory system [64]. The concentration of total SCFAs was enhanced after *E. faecalis* EF-1 intervention, primarily including acetic acid, propionic acid, butyric acid, nonanoic acid, hexanoic acid, heptanoic acid, isobutyric acid, isovaleric acid, capric acid, and octoic acid, indicating that SCFA production was promoted by the gut microbiota.

## 5. Conclusions

In this study, the strain *E. faecalis* EF-1 demonstrated favorable gastrointestinal tolerance, in vitro lipid-lowering efficacy, fatty acid absorption, BSH activity, α-glucosidase inhibitory effects, and antibacterial properties, along with safety. Moreover, in vivo experimental results indicated that *E. faecalis* EF-1 could reduce body weight and ameliorate conditions such as organomegaly, dyslipidemia, and hepatic steatosis, thereby alleviating obesity associated with a HFD. Additionally, *E. faecalis* EF-1 led to a decrease in the *Firmicutes*/*Bacteroidota* ratio and an upsurge in the abundance of SCFA-producing bacteria, effectively ameliorating dysbiosis of gut microbiota and enhancing the metabolic levels of SCFAs. Consequently, *E. faecalis* EF-1 holds promise as a functional food ingredient for combating obesity and reducing lipid levels.

Meanwhile, there are some limits in this research that warrant attention and should be addressed in subsequent studies. Initially, the research was limited to a mouse model, which restricts the extrapolation of findings to human applications. It is essential to conduct additional clinical trials with human subjects to confirm the safety, effectiveness, and dose–response correlations of *E. faecalis* EF-1 for the treatment of obesity and dysregulations in lipid metabolism. Additionally, employing molecular and cellular techniques could facilitate the identification of specific molecular pathways and targets that are influenced by the administration of *E. faecalis* EF-1. Subsequent research efforts should focus on the long-term effects of *E. faecalis* EF-1 and the potential for combination with other dietary supplements, with the aim of mitigating obesity and lipid metabolism disorders.

## Figures and Tables

**Table 1:** The survival results for *E. faecalis* EF-1 in the presence of artificial GI tract juice and bile salts.

Condition	Culture Time/h	Cell No. (log CFU/mL)	Survival Rate/%
Simulated gastric juice	0	5.17 ± 0.55 ^c^	
	1.5	5.33 ± 1.35 ^c^	103.23 ± 26.14 ^c^
	3	5.67 ± 0.85 ^c^	109.68 ± 16.46 ^c^
Simulated intestinal juice	0	4.87 ± 1.10 ^c^	
	2	9.6 ± 1.31 ^c^	197.25 ± 26.87 ^c^
	4	31.67 ± 11.93 ^a^	650.64 ± 245.13 ^b^
	8	43.3 ± 2.52 ^a^	890.35 ± 51.71 ^a^

Notes: The presence of distinct lowercase letters (^a^, ^b^, ^c^) across the columns denotes a statistically significant result with *p* < 0.05.

## Data Availability

The original contributions presented in the study are included in the article, further inquiries can be directed to the corresponding authors.
